# Identification of the Transcriptional Regulator NcrB in the Nickel Resistance Determinant of *Leptospirillum ferriphilum* UBK03

**DOI:** 10.1371/journal.pone.0017367

**Published:** 2011-02-28

**Authors:** Tao Zhu, Jian Tian, Shuangyu Zhang, Ningfeng Wu, Yunliu Fan

**Affiliations:** Biotechnology Research Institute, Chinese Academy of Agricultural Sciences, Beijing, China; University of South Florida College of Medicine, United States of America

## Abstract

The nickel resistance determinant *ncrABCY* was identified in *Leptospirillum ferriphilum* UBK03. Within this operon, *ncrA* and *ncrC* encode two membrane proteins that form an efflux system, and *ncrB* encodes NcrB, which belongs to an uncharacterized family (DUF156) of proteins. How this determinant is regulated remains unknown. Our data indicate that expression of the nickel resistance determinant is induced by nickel. The promoter of *ncrA*, designated *pncrA*, was cloned into the promoter probe vector pPR9TT, and co-transformed with either a wild-type or mutant nickel resistance determinant. The results revealed that *ncrB* encoded a transcriptional regulator that could regulate the expression of *ncrA*, *ncrB*, and *ncrC*. A GC-rich inverted repeat sequence was identified in the promoter *pncrA*. Electrophoretic mobility shift assays (EMSAs) and footprinting assays showed that purified NcrB could specifically bind to the inverted repeat sequence of *pncrA in vitro*; this was confirmed by bacterial one-hybrid analysis. Moreover, this binding was inhibited in the presence of nickel ions. Thus, we classified NcrB as a transcriptional regulator that recognizes the inverted repeat sequence binding motif to regulate the expression of the key nickel resistance gene, *ncrA*.

## Introduction

Metals are essential cofactors for many enzymes in bacterial cells. Nevertheless, many metals become toxic at high ion concentrations, because unlike toxic organic compounds, metals cannot be degraded or modified [1]. The nickel ion, like other metal ions, is essential for bacterial metabolism and becomes toxic at high intracellular concentrations [2]. For example, *Escherichia coli* can endure the presence of no more than 2 mM Ni^2+^ in culture media [3]. Nickel resistance in bacteria is accomplished principally by an operon-encoded and energy-dependent specific efflux system that pumps Ni^2+^ out of the cell, thereby lowering the intracellular concentration [4], [5]. Several nickel-resistant bacteria have been isolated from heavy metal-contaminated locations, and their nickel-resistance systems have been identified. Among these, the best characterized include CnrCBA (Co^2+^ and Ni^2+^ resistance) and NccCBA (Ni^2+^, Co^2+^, and Cd^2+^) of *Cupriavidus metallidurans* CH34 [6], [7], [8], the *nre* and *ncc* determinant (Ni^2+^, Co^2+^, and Cd^2+^) of *Achromobacter xylosoxidans* 31A [9], [10], and the CznCBA efflux system (Co^2+^, Zn^2+^, and Ni^2+^) of *Helicobacter pylori* [11]. A number of new efflux proteins have been identified; for example, RcnA of *E. coli* (Ni^2+^ and Co^2+^ resistance) [12], [13], the *cnr*-like operon of *Comamonas* sp. [14], and *mrdH* of *Pseudomonas putida* (Ni^2+^ and Co^2+^)[15].

In a previous study, we identified a metal-resistant bacterium, *L. ferriphilum* UBK03, and cloned its nickel resistance determinant, including the *ncrA*, *ncrB*, *ncrC*, and *ncrY* genes. *L. ferriphilum* is a genus of iron-oxidizing bacteria which play an important role in the industrial bioleaching and biooxidation [15], [16], [17], [18]. The *ncrA* and *ncrC* genes encode two membrane proteins that together form an efflux system [3]. NcrB is a cytoplasmic, histidine-rich, 89-amino acid (aa) protein of unknown function (Pfam accession no. PF02583) [19]. It contains a conserved 85-aa domain of unknown function (DUF), DUF156, which contains two conserved cysteines and one conserved histidine residue [20]. Similarity analysis revealed that the protein was widely distributed in bacteria [21]. NcrB has been proposed to be a regulator of gene expression [22].

As we know, some nickel responsive regulators (RcnR in rcnR-rcnA efflux system from *Escherichia coli* [12], [23], NikR from *Helicobacter pylori* [24], [25] and Nur from *Streptomyces coelicolor* [26]) have been well identified and characterized. However, the protein NcrB has no apparent sequence similarity to these known transcript regulators.

In this study, we aimed to elucidate the function of NcrB. Using various approaches, we determined that NcrB binds to an inverted repeat sequence within the *pncrA* promoter and represses transcription of *ncrA*, a key gene for bacterial resistance to nickel. Furthermore, NcrB-mediated transcriptional repression was inhibited in the presence of Ni^2+^.

## Results

### Induction of nickel resistance

**Figure 1:**
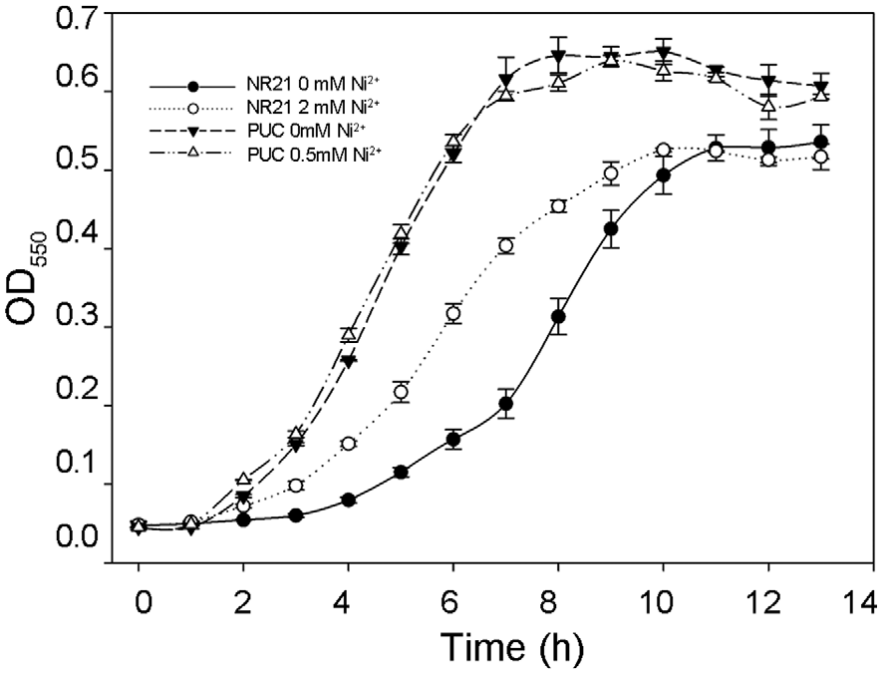
Growth curve of *E. coli* harboring pNR21 or pUC19 plasmid in medium containing the NiCl_2_ either induced or not induced by NiCl_2_. Filled triangles, *E.coli* harboring pUC19 (PUC) without induced; open triangles, *E. coli* haboring pUC19 (PUC) induced by 0.5 mM Ni^2+^; Filled circles, NR21 without induced; open circles, NR21 induced by 2 mM Ni^2+^. *E. coli* was grown at 37°C containing 4 mM NiCl_2_ (*E*. *coli* NR21) or 1 mM (*E*. *coli* PUC) and the optical density was monitored at 550 nm.

The expression of most bacterial metal resistance systems is induced [27]. To investigate whether the nickel resistance determinant of *L*. *ferriphilum* UBK03 [3] is also inducible, the effect of nickel on *E. coli* NR21 growth was assessed. When non-induced *E. coli* NR21 was exposed to 4 mM NiCl_2_, there was a growth delay of 2 h compared with *E. coli* NR21 induced with 2 mM NiCl_2_, although the growth yield was unaffected (Fig. 1).

**Figure 2:**
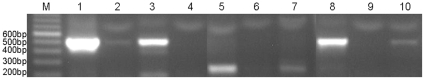
Transcription of *ncrA*, *ncrB*, and *ncrC* is induced by 4 mM NiCl_2_. Lanes 1-4: PCR amplification (502 bp) of *ncrA* from genomic DNA using primers RT-*nrcAF* and RT-*nrcAR* (lane 1); cDNA from non-induced cultures (lane 2); cDNA from nickel-induced cultures (lane 3); and RNA from nickel-induced cultures (lane 4). Lanes 5-7: PCR amplification (270 bp) of *ncrB* from genomic DNA with primers RT-*nrcBF* and RT-*nrcBR* (lane 5); cDNA from non-induced cultures (lane 6); and cDNA from nickel-induced cultures (lane 7). Lanes 8–10: PCR amplification (523 bp) of *ncrC* from genomic DNA with primers RT-*nrcCF* and RT-*nrcCR* (lane 8); cDNA from non-induced cultures (lane 9); and cDNA from nickel-induced cultures (lane 10).

RT-PCR was conducted to confirm that the nickel resistance system is inducible. The transcription of *ncrA*, *ncrB*, and *ncrC* was upregulated in the presence of Ni^2+^ (Fig. 2). Moreover, RT-QPCR revealed that the presence of Ni^2+^ in culture medium resulted in a 10-fold increase in *ncrA* transcription. These data suggest that Ni^2+^ induces transcription of the nickel resistance system.

### Construction of promoter-lacZ fusion plasmids

**Figure 3:**
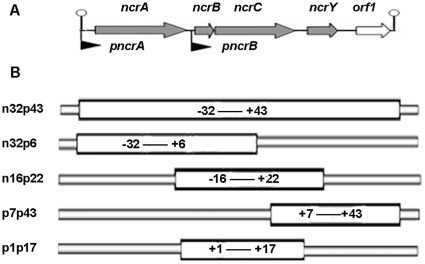
Structures of the promoters. (**A**) Schematic of the locations of *pncrA* and *pncrB*. (**B**) Schematic of *pncrA* (n32p43) and partial regions of the promoter. Numbers indicate positions relative to the transcription start site.

**Figure 4:**
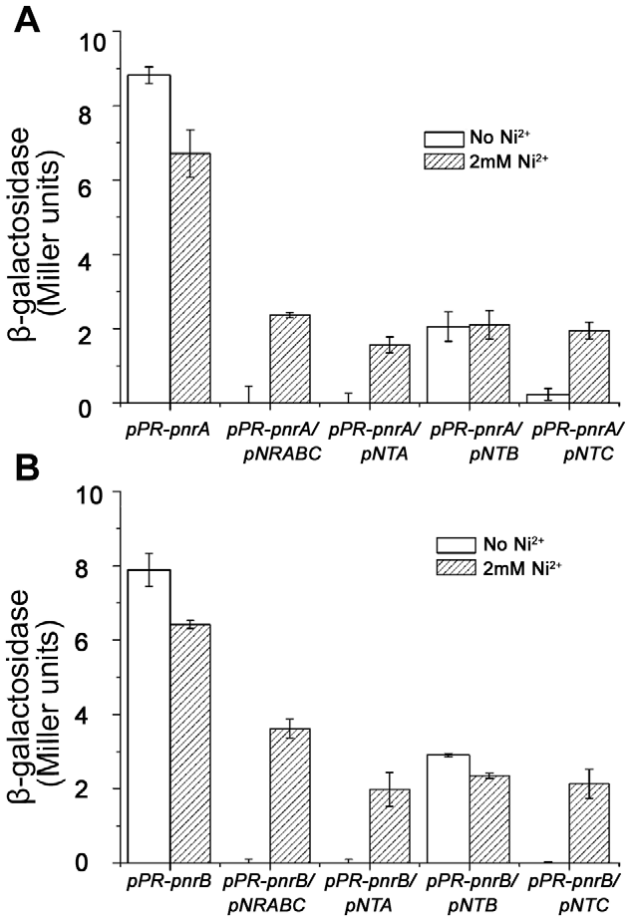
Determination of promoter activity. (**A**) pPR-*pncrA* alone or with pNRABC, pNTA, pNTB, or pNTC was used to transform *E. coli* JM109 cells. Transformants were cultured under non-inducing (open bars) or 2 mM NiCl_2_ (shaded bars) conditions. (**B**) pPR-*pncrB* alone or with pNRABC, pNTA, pNTB, or pNTC was used to transform *E. coli* JM109 cells. Transformants were cultured under non-inducing (open bars) or 2 mM NiCl_2_ (shaded bars) conditions. Error bars indicate the SD from four independent experiments.

Analysis of the sequence immediately upstream of *ncrA* and *ncrB* revealed the presence of two promoters (*pncrA* and *pncrB*, Fig. 3). These regions were inserted into the upstream of *lacZ* in pPR9TT, a low copy-number *lacZ*-based promoter probe plasmid [28] to construct the plasmids pPR-pncrA and pPR-pncrB. These two plasmids and pPR9TT (negative control) were transformed into *E. coli* JM109, respectively. No β-galactosidase activity was detected with pPR9TT in *E. coli* JM109 (data not shown), whereas about 9 Miller units of β-galactosidase activity were detected in with pPR-pncrA and pPR-pncrB (Fig. 4). These data indicate that pPR-pncrA and pPR-pncrB acted as the constitutive promoters in the absence of the nickel resistance genes (*ncrA*, *ncrB,* and *ncrC*).

### Activity of the promoters with different nickel resistant genes

The promoter probe plasmids (pPR-pncrA and pPR-pncrB) and pNRABC, which contains the nickel resistance genes (*ncrA*, *ncrB*, and *ncrC*) [3], were co-transformed into *E. coli* JM109, and transformants were selected using 50 µg/mL ampicillin and 4 mM Ni^2+^. Both *pncrA* and *pncrB* were induced in the presence of Ni^2+^. The results further suggested that NcrA, NcrB, or NcrC may contain a transcriptional regulator. Then, *E. coli* JM109 cells were transformed with the plasmid pPR-pncrA or pPR-pncrB alone, or co-tranformed with the plasmid pNTA, pNTB, or pNTC (which contained the inserted mutations in *ncrA*, *ncrB*, or *ncrC* by tetracycline box insertion) [3]. Transformants were selected using 50 µg/mL ampicillin and 50 µg/mL tetracycline. Both promoters were constitutively active when co-transformed with mutated *ncrB*, but not with mutated *ncrA* or *ncrC* (Fig. 4). These data suggest that the NcrB protein functions as a transcriptional regulator to regulate the activity of *pncrA* and *pncrB*.

### Interaction of NcrB with the promoter pncrA

**Figure 5:**
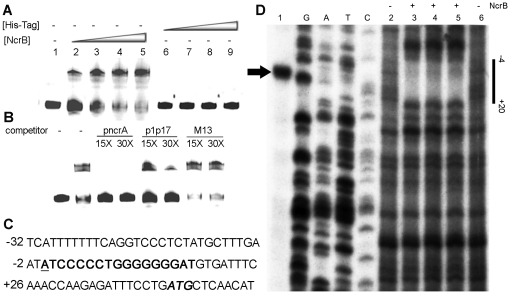
The interaction between NcrB and pncrA *in vitro*. Electromobility shift assays (A–B). (**A**) The *pncrA* fragment was incubated with His_6_-tagged NcrB or the His_6_-tag at the indicated concentrations. Lane 1, no protein; lane 2, 0.2 µM NcrB; lane 3, 0.4 µM NcrB; lane 4, 0.8 µM NcrB; and lane 5, 1.6 µM NcrB; lane 6, 0.2 µM His_6_-tag; lane 7, 0.4 µM His_6_-tag; lane 8, 0.8 µM His_6_-tag; and lane 9, 1.6 µM His_6_-tag. (**B**) The fragment was incubated with both 0.8 µM NcrB and unlabeled competitor at the fold-concentrations indicated above the lanes. (C) Sequence of the promoter *pncrA*. The GC-rich inverted repeat sequence (bold), the transcription start site of *ncrA* (bold and underlined) and the potential translation initiation codon (bold and italic) are indicated along the sequences. (D) The transcription start site of *ncrA* and Dnase I footprint of NcrB on pncrA. Lane 1, the arrowhead indicates the transcription start point. Lane G, A, T and C indicate the nucleotide sequence ladders of *pncrA*. Lane 2 and 6, DNase I digestions as a control (No NcrB). Lanes 3–5, purified NcrB protein was added to the final concentration from (0.1 µM, 0.2 µM and 0.4 µM).

The transcription start point of *ncrA* was localized at position 44 nt upstream of the potential start codon (ATG) of *ncrA* by the high-resolution S1 nuclease mapping (Figure 5D). As shown in Fig. 5D, a high GC content and inverted repeat sequence (p1p17) was identified at the downstream of the transcription start point. The possibility of a direct interaction of NcrB with the putative operator in *pncrA* was assessed *in vitro* by EMSA. The *ncrB* gene was ligated into the expression plasmid pET30a(+), purified and assessed by SDS-PAGE. The *pncrA* fragment was labeled using infrared dye-labeled M13 oligos and purified [29]. The EMSA results showed that NcrB caused a slower movement of labeled *pncrA*, indicating that NcrB binds to *ncrA* (Fig. 5A). Moreover, given the large excess of competitor DNA [poly (dI-dC)] or M13 primer in the binding mix, NcrB–*pncrA* binding must be specific. Binding was significantly reduced in the presence of unlabeled *pncrA* or the 17-bp inverted repeat. Thus, NcrB could bind *pncrA* at the 17-bp inverted repeat region.

The DNase I footprinting experiment was also used to determine the binding sites of NcrB in the promoter region of *pncrA*. As shown in Fig. 5D, a protected region from positions −4 to +25 relative to the transcription start point of ncrA was detected. Moreover, the high GC content and inverted repeat sequence was also located at the protected region. These results indicated that the protein NcrB could bind to the 17-bp inverted repeat region *in vitro*.

### *In vivo* binding of NcrB to promoter regions

A bacterial one-hybrid system [30], [31] was used to test NcrB binding to promoter regions *in vivo*. For analysis of the NcrB binding site, the sequence of the promoter region (n32p43) was divided into three overlapping fragments (n32p6, n16p22, and p7p43), as shown in Fig. 3B. Five different overlapping fragments and the inverted repeat region (Fig. 3B) from *pncrA* were inserted respectively into the prey plasmid pH3U3, which has two reporter genes (yeast *HIS3* and *URA3*). These plasmids were used to transform *E. coli* US0hisB-pyrF- cells, and transformants were screened in medium containing 4.5 mM 5-FOA, 30 µg/mL chloramphenicol, and 20 µg/mL tetracycline. All of the transformants were able to grow on YM plates containing 4.5 mM 5-FOA, indicating that the *URA3* reporter gene in the prey plasmid was not expressed. Thus, these regions of *pncrA* did not self-activate reporter gene expression.

**Figure 6:**
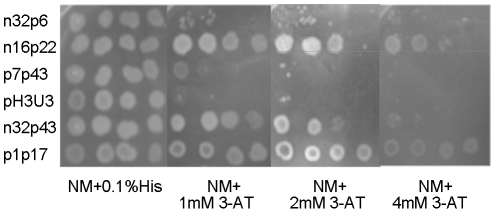
Physical interaction between *pncrA* and NcrB. A bacterial one-hybrid system was used to detect *pncrA*-protein interactions. The growth rates of cells containing different bait/prey combinations were examined under positive selection. Each population of cells was serially diluted in ten-fold steps (left to right) and plated on NM minimal medium containing various 3-amino-1,2,4-triazole (3-AT) concentrations, as indicated below the panel.

The *ncrB* gene was then inserted into the bait plasmid pB1H1, forming pB1H1-*ncrB*. This bait plasmid and the prey plasmids were used to co-transform *E. coli* US0hisB-pyrF- cells, and transformants were screened on His-selective plates [30], [31] containing different concentration of 3-AT. Co-transformed strains containing either n16p22 or the inverted repeat region (p1p17) of *pncrA* in the prey plasmid were able to grow on 3-AT plates (Fig. 6). However, the strains that contained the n32p6 or p7p43 region, which did not contained an intact inverted repeat, were not capable of growth on 3-AT plates. These results indicate that NcrB binds directly to the 17-bp (G+C)-rich inverted repeat sequence (5′-ATCCCCCTGGGGGGGAT-3′) in the p1p17 region.

### The effect of Ni^2+^ on the binding between NcrB and the inverted repeat region (p1p17)

**Figure 7:**
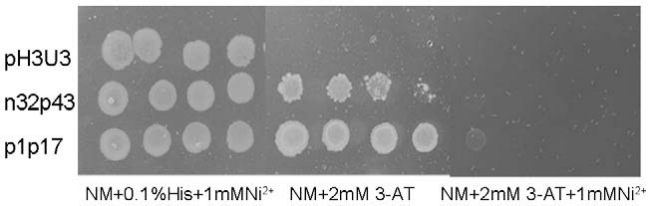
Inhibition of *pncrA* and NcrB binding in the presence of nickel. A bacterial one-hybrid system was used to detect n32p43 (*pncrA*)-protein and p1p17-protein interactions. Growth rates of cells containing different bait/prey combinations were examined under positive selection. Left panel: NM plate supplemented with 0.1% histamine and 1 mM NiCl_2_ as a control; Middle panel: NM plate supplemented with 2 mM 3-AT. Right panel: NM plate supplemented with 2.0 mM 3-AT and 1 mM NiCl_2_. Each population of cells was serially diluted in ten-fold steps (from left to right) and plated.

To test its effect on the binding between NcrB and the inverted repeat region, Ni^2+^ was added to the bacterial one-hybrid system by adding 1 mM Ni^2+^ to the 3-AT selective screening medium plate. As shown in Fig. 7, the strains containing the promoter (n32p43) or inverted repeat region (p1p17) in the prey plasmid grew on the 3-AT selective medium plates, but could not grow on the 3-AT plates containing 1 mM Ni^2+^. The presence of 1 mM Ni^2+^ in the medium did not affect the growth of *E. coli* [3], [9]. Thus, 1 mM Ni^2+^ could disrupt the binding between NcrB and the inverted repeat region (p1p17).

## Discussion

In a previous report, we identified the metal-resistant bacterium *L. ferriphilum* UBK03 and cloned its nickel resistance determinant, which included *ncrA*, *ncrB*, *ncrC*, and *ncrY*. NcrA contains 10 transmembrane helices and is the foundation of the nickel resistance complex [3]. NcrC, similar to NcrA, is a membrane protein belonging to the high-affinity nickel transport protein family (Pfam accession no. PF03824) and contributes significantly to nickel resistance, possibly by chelating nickel cations in the cytoplasm [13], [21], [32].

NcrB is a cytoplasmic, histidine-rich, 89-aa protein of unknown function (Pfam accession no. PF02583). It belongs to the DUF156 group of proteins that are widely distributed in bacteria. Sequence analysis revealed that NcrB contains a leucine zipper domain [33] at the N-terminus. This domain is rich in positively charged amino acid residues, which may contribute to DNA recognition and binding. The C-terminal region is rich in negatively charged amino acid residues, suggesting that this region may be involved in polymerization (Figure S1). Taken together, these features indicated that NcrB may be a transcriptional regulator. Some authors have speculated that NcrB acts as a regulator of gene expression [22]. In the present study, we identified the regulatory function of NcrB and its recognition site.

NcrB regulated the *pncrA* and *pncrB* promoters. NcrB appeared to bind to the GC-rich inverted repeat region of *pncrA*, which is not present in *pncrB* (Fig. 3A). NcrB may recognize another region of *pncrB* and thereby regulate the expression of *ncrB* and *ncrC*. The effect of NcrB on *pncrB* is currently being investigated in our laboratory. Data from a bacterial one-hybrid system indicated that NcrB binds directly to *pncrA*, and this binding was inhibited by 1 mM Ni^2+^. In an EMSA assay to elucidate the effect of Ni^2+^, the Ni^2+^ unexpectedly formed a precipitate in the binding buffer; thus, this assay could not be carried out.

In conclusion, NcrB is a transcriptional repressor of the nickel resistance determinant in *L. ferriphilum* UBK03. In the presence of low concentrations of Ni^2+^, NcrB binds to the inverted repeat region of *pncrA*, thereby repressing its function. However, at high concentrations of Ni^2+^, the repression by NcrB is removed, *pncrA* becomes active, and the *ncrA* nickel resistance gene is expressed.

## Materials and Methods

### Bacterial strains and culture conditions

Table 1 lists the strains and plasmids used in this study. The *E. coli* strains were grown aerobically in Luria-Bertani (LB) medium at 37°C with continuous shaking at 200 rpm. For selection of *E. coli* transformants, ampicillin and kanamycin were added to final concentrations of 100 and 50 µg/ml, respectively. As bacterial one-hybrid system selective media, His-selective (positive) NM medium and 5-FOA-selective (negative) YM medium were used as described previously [30]. Isopropyl-β-D-thiogalactoside (IPTG), o-nitrophenyl-β-D-galactopyranoside (ONPG), amino acids, 5-fluoro-orotic acid (5-FOA), and 3-amino-triazole (3-AT) were purchased from Sigma (St. Louis, MO). Other reagents were of analytical grade and were purchased from JingKeHongDa Biotechnology Co., Ltd, China.

**Table 1:** Bacterial strains and plasmids used in this study.

Strains and plasmids	Relevant genotype or characteristic(s)	Reference or source
***E.coli***		
Trans10	F-Ф80(lacZ) ΔM15ΔlacX74hsdR(rK-mK+)ΔrecA1398endA1tonA	TransGen
BL21(DE3)	F- ompT hsdS (rB - mB-) gal dcm lacY1(DE3)	Novagen
US0	F'episome bearing the lacIq repressorΔhisBΔpyrF	[30], [31]
NR21	*E.coli* JM109 containing pNR21	[3]
PUC	*E.coli* JM109 containing pUC19	Promega
**Plasmids**		
pNR21	4.0-kb HindIII fragment containing *ncrA*, *ncrB*, *ncrC* and *ncrY* from genomic DNA of strain UBK03, Amp^r^	[3]
pPR9TT	Broad-host range *lac*Z promoter probe vector; RK2 replicon; Amp^r^, Cm^r^	[28]
pPR-pncrA	pPR9TT containing insertion in promoter *ncrA*	This study
pPR-pncrB	pPR9TT containing insertion in promoter *ncrA*	This study
pNRABC	pNR21 containing insertion in *ncrA*,*ncrB*,*ncrC*, promoter *ncrA*,and promoter *ncrB*	[3]
pNTA	pNR21 containing insertion in *ncrA*, Amp^r^, Tc^r^	[3]
pNTB	pNR21 containing insertion in *ncrB*, Amp^r^, Tc^r^	[3]
pNTC	pNR21 containing insertion in *ncrC*, Amp^r^, Tc^r^	[3]
pET30a(+)	Km^r^, Expression vector	Novagen
pH3U3	Km^r^, pSC101 origin of replication	[30], [31]
pB1H1	Cm^r^, p15A origin of replication	[30], [31]
pEASY-T3	Amp^r^, Cloning vector	TransGen
pET30-ncrB	BamHI-HindIII fragment containing ncrB inserted into pET30a(+), Km^r^	This study
pB1H1-ncrB	NotI-AvrII fragment containing *ncrB* inserted into pB1H1, Km^r^	This study
n32p43	NotI-EcoRI fragment containing *ncrA* promoter region between positions −32 and +43 inserted into pH3U3	This study
n32p6	NotI-EcoRI fragment containing *ncrA* promoter region between positions −32 and +6 inserted into pH3U3	This study
n16p22	NotI-EcoRI fragment containing *ncrA* promoter region between positions -16 and +22 inserted into pH3U3	This study
p7p43	NotI-EcoRI fragment containing *ncrA* promoter region between positions +7 and +43 inserted into pH3U3	This study
p1p17	NotI-EcoRI fragment containing *ncrA* promoter region between positions +1 and +17 inserted into pH3U3	This study

### Effect of Ni^2+^ on *E*. *coli* NR21 and *E*. *coli* PUC

*E. coli* was cultivated overnight in LB with or without 2 mM Ni^2+^ (*E*. *coli* NR21) or 0.5 mM Ni^2+^ (*E*. *coli* PUC), diluted 100-fold into fresh LB medium containing 4 mM NiCl_2_ (*E*. *coli* NR21) or 1 mM (*E*. *coli* PUC), and grown at 37°C with shaking at 200 rpm. The optical density at 550 nm was monitored hourly for 13 h.

### RT-PCR and quantitative RT-PCR (QRT-PCR)

The effect of Ni^2+^ on *ncrA*, *ncrB,* and *ncrC* expression was assessed by RT-PCR. Cells were grown in the presence or absence of 2 mM Ni^2+^. Total RNAs were extracted using an RNAprep pure bacteria kit (TianGen, China) according to the manufacturer's instructions, and cDNA was synthesized from 4 µg of total RNA using Moloney murine leukemia virus reverse transcriptase (TianGen, China) at 42°C for 50 min. PCR was performed for 30 cycles under the following conditions: denaturation at 94°C for 15 s, annealing at 55°C for 20 s, and extension at 72°C for 20 s. PCR products were analyzed by agarose gel electrophoresis. QRT-PCR was performed using a real-time PCR system (Bio-Rad). SYBR Green master mix (Toyoto, Japan) was used to amplify DNA under the following conditions: initial denaturation at 95°C for 3 min, followed by 40 amplification cycles (15 s at 95°C, 20 s at 55°C, and 20 s at 72°C). Melt curve data were collected using 10-s cycles (55°C for 80 cycles). Duplicate cycle threshold (CT) values were analyzed by the comparative 2^-ΔΔCT^ method [34]. The relative amount of target mRNA was obtained by normalizing to an ampicillin resistance reference gene.

### Bacterial one-hybrid analysis

Bacterial one-hybrid analysis was performed as described previously [30], [31] with some modifications. High-efficiency electrocompetent XL1-blue *E. coli* cells were substituted for *E. coli* Trans10 (TransGen, China). The promoter *ncrA* (pncrA) and its deletions were amplified by PCR with primers containing *Not*I and *Eco*RI sites, and then cloned into the *Not*I*–Eco*RI sites of the reporter plasmid pH3U3. *NcrB* was amplified by PCR and inserted into the *Not*I and *Avr*II sites of the bait plasmid pB1H1. The constructs were verified by sequencing at the State Key Laboratory of Crop Genetic Improvement, Chinese Academy of Agricultural Sciences, Beijing, China. The two plasmids were used to co-transform the selection strain US0 by electroporation [31], and co-transformants were selected using medium that contained 3-AT, chloramphenicol (30 µg/mL), kanamycin (25 µg/mL), and tetracycline (20 µg/mL). Self-activation experiments were performed using selective medium containing 4.5 mM 5-FOA, chloramphenicol (30 µg/mL), and tetracycline (20 µg/mL).

### Expression and purification of His-tagged NcrB protein

An *ncrB*-containing DNA fragment was amplified by PCR using plasmid pNR21as the template and primers pET-ncrB_R and pET-ncrB_F (Table S1). The PCR product was purified using a gel extraction kit (TianGen, China), digested with *Bam*HI and *Hin*dIII, and ligated into pET-30a(+) vector. Insertion was confirmed by sequencing. For protein expression, the plasmid was used to transform *E. coli* BL21 (DE3) cells. Transformants were cultured in LB medium (100 mL), and IPTG (final concentration, 1 mM) was added when the A_600_ of the culture reached 0.6. After incubation for 12 h at 16°C, the cells were harvested by centrifugation and washed with lysis buffer (20 mM Tris-HCl, pH 8.0; 4°C). As the N-terminus of recombinant NcrB was fused to a His_6_ tag, NcrB was purified using a Ni-NTA His-bind™ resin column (Novagen, San Diego, CA) according to the manufacturer's instructions. The column eluate was desalted with lysis buffer and stored at −20°C until required. Purified protein was assessed by sodium dodecyl sulfate-polyacrylamide gel electrophoresis (SDS-PAGE). Protein concentrations were quantified using a Bio-Rad protein assay kits II (Bio-Rad Laboratories (Beijing) Ltd. China).

### Electrophoretic mobility shift assay (EMSA)

DNA fragments containing different promoter fragments were prepared by PCR using primers ProAF and ProAR (Table S1). The *pncrA* fragment was labeled using infrared dye-labeled M13 oligos and purified as described previously [29]. Each reaction mixture (20 µL) contained infrared dye-labeled probe and His-NcrB in buffer (10 mM Tris, pH 7.5, 50 mM NaCl, 1 mM dithiothreitol, 0.25% Tween 20, 5 mM MgCl_2_, and 1 µg of poly(dI-dC). The mixture was incubated for 20 min at 37°C in the dark, followed by electrophoresis in an 8% non-denaturing polyacrylamide gel in 0.5× TBE buffer, at 80 V for 50 min with a mini-protein electrophoresis system. The mobility positions of the labeled products in the gel were detected using the Odyssey software package (LI-COR Biosciences UK Ltd., Cambridge, UK).

### S1 nuclease protection analysis

Total RNAs of NR21 induced by 4 mM NiCl_2_ were extracted using an RNAprep pure bacteria kit (TianGen, China) according to the manufacturer's instructions. The S1 nuclease protection analysis were performed as described previously [35], [36]. The *pncrA* probe was prepared by PCR using the unlabeled primer pncrA-map-F and the 5′-end [γ-^32^P] ATP-labeled primer pncrA-map-R. A DNA sequencing ladder was generated using the same labeled primer with an fmol DNA cycle sequencing kit (Promega). The protected fragments were analyzed on a 6.0% polyacrylamide gel containing 7 M urea.

### DNase I footprinting assays

In order to determine the NcrB binding sites in *pncrA* promoter region, DNase I footprinting assays were carried out as described previously [35], [36]. The probe was prepared by labelling the 5′ end of *pncrA* antisense stand using primers pncrA-map-F and pncrA-map-R. The primer pncrA-map-R was ^32^P-labelled with T4 polynucleotide kinase before PCR. The footprinting reaction mixture contained 40 000 cpm of ^32^P-labelled DNA probe, different concentrations of His-NcrB, 10 mM Tris-HCl (pH 7.5), 2 mM DTT, 0.5 mg/mL calf BSA and 5% glycerol in a total volume of 50 µL. After incubation of the mixture at 25°C for 30 min, 0.4 U DNase I (Promega) was added to the binding mixture. It was further incubated at 25°C for 70 seconds and was stopped by the addition of 50 µL stop solution (20 mM EDTA, pH 8.0) and 100 ml phenol-chloroform (1∶1, v/v). DNA fragments in the aqueous phase were precipitated by adding 10 µL ammonium acetate (3 M), 2 µL glycogen (10 mg/mL) and 2.5 vol ethanol, washed with 75% ethanol, dried and directly suspended in 10 ml of 90% formamide-loading gel buffer (10 mM Tris-HCl, pH 8.0, 20 mM EDTA, pH 8.0, 0.05% bromophenol blue, 0.05% xylene cyanol). Samples were then denatured at 95°C for 10 min and 2 µL of each sample was loaded on a 6% polyacrylamide–urea gel. The sequence ladder was same to the ladder in the S1 nuclease protection analysis. After electrophoresis, the gels were dried and exposed to Kodak X-ray film.

### Construction of pncrA-lacZ and pncrB-lacZ fusions

The plasmid pPR9TT was used to assess *pncrA* and *pncrB* function. The promoter regions *pncrA* and *pncrB* were amplified from pNR21 using the primers pPR-pncrA_F, pPR-pncrA_R, pPR-pncrB_R, and pPR-pncrB_F (Table S1). The PCR-amplified DNA fragments were digested with *Xho*I and *Pst*I and ligated into *Xho*I- and *Pst*I-cleaved pPR9TT, yielding pPR-pncrA and pPR-pncrB. Correct gene insertion was confirmed by DNA sequencing, performed at the State Key Laboratory of Crop Genetic Improvement, Chinese Academy of Agricultural Sciences.

### β-galactosidase assay

β-Galactosidase activity was measured as described by Miller [37] and expressed in Miller units. The data presented are the results from at least three independent experiments, with a standard deviation of 10%.

## Supporting Information

Table S1 Sequence of oligonucleotide primers used in this study.
(DOC)

Figure S1 The leucine zipper structure of NcrB. The residues below the triangle were the leucines in the leucine zipper structure.
(DOC)
