# Supplementary material for: Identification of the Transcriptional Regulator NcrB in the Nickel Resistance Determinant of Leptospirillum ferriphilum UBK03
Source: PLoS One. 2011 Feb 28;6(2):e17367. doi: 10.1371/journal.pone.0017367 (PMC3046157; doi:10.1371/journal.pone.0017367)
Supplement: Table S1 — Sequence of oligonucleotide primers used in this study. (DOC) [file pone.0017367.s001.doc]

**Table S1. Sequence of oligonucleotide primers used in this study**

| **Primers** | **Sequences (5’-3’)^a^** |
| --- | --- |
| RT-nrcAF  RT-nrcAR  qRT-ncrAF  RT-nrcBF  RT-nrcBR  RT-ncrCF  RT-ncrCR  qRT-ampF  qRT-ampR  n32p43F  n32p43R  n32p6F  n32p6R  n16p22F  n16p22R  p7p43F  p7p43R  p1p17F  p1p17R  OH-ncrBF  OH-ncrBR  ProAF  ProAR  ProBR  ProBF  pPR-pncrA_F  pPR-pncrA_R  pPR -pncrB_R  pPR-pncrB_F  pET-ncrB_F  pET-ncrB_R  pncrA-map-F  pncrA-map-R | GTACCGCGCTGGCTATCAAA  CTGGGCGTTTTCAGGAAGAT  CGCTGTCTCTGTCACGTCTGG  ATGACCGTTCATGCATCACAC  TTAAAGATATTTAGTTATCTC  CTGAATCAGCCGAACCCTGGT  TGCGCTTTTTCGTGAGCATCC  AATTGTTGCCGGGAAGCTAGAGTAAGTA  GCCGCATACACTATTCTCAGAATGACTTG  cat GCGGCCGC TCATTTTTTTCAGGTC  cat GAATTC CAGGAAATCTCTTGGTTTGAAAT  cat GCGGCCGCTCATTTTTTTCAGGTC  cat GAATTC GGGGATATTCAAAGCA  cat GCGGCCGC CCTCTATGCTTTGAAT  cat GAATTC ATCACATCCCCCCCAG  cat GCGGCCGC CTGGGGGGGAT  cat GAATTC CAGGAAATCTCTTGGT  cat GCGGCCGC ATCCCCCTGGGGGGGATGAATTCatg  cat GAATTC ATCCCCCCCAGGGGGATGCGGCCGCatg  cat GCGGCCGCC ATGACCGTTCATGCATCACA  cat CCTAGG TTAAAGATATTTAGTTATCTCT  CACGACGTTGTAAAACGAC TCATTTTTTTCAGGTC  GGATAACAATTTCACACAGG CAGGAAATCTCTTG  CACGACGTTGTAAAACGAC TTTCCCAAAAAAAGAC  GGATAACAATTTCACACAGG GGTTTATCCTCTC  ataCTCGAGTGTGATTGCCCGATTATCTC  ataCTGCAGGACATCAGGAAATCTCTTGGTT  ataCTCGAG TTACATCACGCTGGTTGTGC  ataCTGCAGGA CATGGTTTATCCTCTCCAGG  ttaGGATCCATGACCGTTCATGCATCACA  tatAAGCTTTGTTTCGCTTGCCTTAAAGA  CTGCCATCCAGGTGTTCTACGAGAG  TGTCACCTGCGAGATCGTAGGCCAG |

Restriction enzyme sites are shown in bold and underlined.
