# Supplementary material for: Identification of the Transcriptional Regulator NcrB in the Nickel Resistance Determinant of Leptospirillum ferriphilum UBK03
Source: PLoS One. 2011 Feb 28;6(2):e17367. doi: 10.1371/journal.pone.0017367 (PMC3046157; doi:10.1371/journal.pone.0017367)
Supplement: Figure S1 — The leucine zipper structure of NcrB. The residues below the triangle were the leucines in the leucine zipper structure. (DOC) [file pone.0017367.s002.doc]

Figure S1. The leucine zipper structure of NcrB. The residues below the triangle were the leucines in the leucine zipper structure.

AAAAAAAA10AAAAAAAA20AAAAAAAA30AAAAAAAA40AAAAAAAA50AAAAAAAA60

ATGACCGTTCATGCATCACACCCTGACATTATTAAAAGACTTAAACGTGCCGCAGGGCAC

MTGTTGVTGHTGATGSTGHTGPTGDTGITGITGKTGRTGLTGKTGRTGATGATGGTGHTG

AAAAAAAA70AAAAAAAA80AAAAAAAA90AAAAAAA100AAAAAAA110AAAAAAA120

CTGAAAAGTACCATCCAGATGCTCGAAGATGAGAAAGCATGTCTGGATATCGCCCAGCAA

LTGKTGSTGTTGITGQTGMTGLTGETGDTGETGKTGATGCTGLTGDTGITGATGQTGQTG

AAAAAAA130AAAAAAA140AAAAAAA150AAAAAAA160AAAAAAA170AAAAAAA180

CTGCACGCTGTGGAAAAGGCGATAACGAATGCGAAGCGTACCTTAATTCATGACCATCTG

LTGHTGATGVTGETGKTGATGITGTTGNTGATGKTGRTGTTGLTGITGHTGDTGHTGLTG

AAAAAAA190AAAAAAA200AAAAAAA210AAAAAAA220AAAAAAA230AAAAAAA240

GATCACTGCCTGGAGGATGCCCTTCATGCTGACCACACGGAGTCTGATAAGACGCTTAGC

DTGHTGCTGLTGETGDTGATGLTGHTGATGDTGHTGTTGETGSTGDTGKTGTTGLTGSTG

AAAAAAA250AAAAAAA260AAAAAAA270A

GAATTTAAAGAGATAACTAAATATCTT

ETGFTGKTGETGITGTTGKTGYTGLTG
